# A New Assessment for Activities of Daily Living in Spanish Schoolchildren: A Preliminary Study of its Psychometric Properties

**DOI:** 10.3390/ijerph17082673

**Published:** 2020-04-13

**Authors:** Sabina Barrios-Fernández, Margarita Gozalo, Andrés García-Gómez, Dulce Romero-Ayuso, Miguel Ángel Hernández-Mocholí

**Affiliations:** 1 Department of Medical-Surgical Therapeutics, University of Extremadura, 10003 Cáceres, Spain; 2 Department of Psychology and Anthropology, University of Extremadura, 10003 Cáceres, Spain; mgozalo@unex.es; 3 Department of Education Sciences, University of Extremadura, 10003 Cáceres, Spain; agarcil9@unex.es; 4 Department of Physiotherapy, University of Granada, 18071 Granada, Spain; dulceromero@ugr.es; 5 Department of Didactics of Musical, Plastic and Corporal Expression, University of Extremadura, 10003 Cáceres, Spain; mhmocholi@unex.es

**Keywords:** activities of daily living, executive function, emotional regulation, assessment, child

## Abstract

Background: Activities of daily living (ADL), which are divided into basic (BADL) and instrumental (IADL), allows us to survive and to live in the society. Cognitive skills are a key aspect in BADL outcomes. After reviewing existing BADL tools for Spanish schoolchildren, issues such as not covering the full age range or not having a BADL-centred vision were found. We aim to develop a new tool for BADL assessment in Spanish schoolchildren. Methods: The new tool was administered to 375 participants (47.2% boys and 52.8% girls) from 6 to 12 years of age. Analyses were carried out to find out the structure (semiconfirmatory factor analysis) and internal consistency (ordinal alpha) of BADL. Results: Four scales formed the instrument (Eating, Personal Hygiene, Getting Dressed, and General Functioning) with an interpretable solution of 12 factors (Manual Dexterity, Proprioception, Oral Sensitivity, Good Manners, Hygiene and Grooming, Toileting Needs Communication, Bladder and Bowel Control, Showering, Independent Dressing Tasks, Full Dressing, Executive Functions, and Self-Regulation) with 84 items + 6 qualitative items for girls. The reliability values obtained were acceptable (.70–.88). Conclusions: The tool seems to be a practical and reliable instrument to assess BADL and cognitive skills during BADL in Spanish schoolchildren.

## 1. Introduction

Activities of daily living (ADL) are a set of activities performed by humans both for survival and for living in the society [1]. Depending on their objectives, complexity, and level of privacy, ADL are divided into basic ADL (BADL) or instrumental ADL (IADL). On the one hand, BADL include care activities for basic needs, are considered to be universal, and require minimal cognitive effort, like bathing, toileting and personal hygiene, dressing, feeding, functional mobility, taking care of own devices, and sexual activity. On the other hand, IADL refer to activities for home management or taking care of others, depend on the culture, and require a more considerable cognitive effort [2,3], such as communication management, community mobility, taking care of others, health maintenance, money, shopping, home management, and involvement in spiritual activities [1,4,5]. Humans learn ADL throughout their whole life, but childhood and early adolescence are critical periods. Good performance of these activities is essential to be functional, to develop an independent and self-determined life, and for social inclusion [6,7,8,9,10,11]. Under the occupational therapy (OT) practitioners’ view, BADL are one of the human occupations. Occupations, Client Factors, Performance Skills, Performance Patterns, Context and Environment interact with each other and with the person, so all of them are critical topics for OT [1], as shown in Table 1.

Within the Performance Skills group, some neurobiological processes are relevant for ADL performance [12,13]: executive function (EF) and self-regulation (SR). EF belongs to the group of cognitive processes related to behavior, including acceptably solving internal and external problems [14,15], and plays an essential role in facing new situations [16]. EF should include three main domains: updating/working memory (keeping online relevant information during a task), inhibition (stopping predominant thoughts or actions), and flexibility (being able to change one’s perspective) [17,18]. Proper EF functioning is necessary for adaptive ADL performance [19,20,21], as we continually plan and sequence, solve problems [22,23], keep organized and focused during daily activities. SR refers to being able to manage thoughts, attention, feelings, responses related to stimuli [24] and behaviors to enable goal-directed actions [25]. SR is necessary for successful adaptation to the environment, including the performance of occupations such as eating, dressing, bathing, sleeping, and learning [26]. Some of the EF processes support SR, so EF should be a necessary but not sufficient condition for good SR performance [27,28].

Clinical intervention in OT includes assessment, planning, and treatment. With regards to evaluation, sometimes OT practitioners use tools from other disciplines. This is not a problem itself, except if theoretical bases of the tool can implicate results of the occupational diagnosis [29,30]. If we compare ADL and adaptive behavior concepts, then we can see that they are not the same: ADL refer to people’s daily self-care activities, come from the OT field, and include BADL and IADL. Adaptive behavior comes from psychology and intellectual disability, and it is defined as appropriate behaviors that people perform to live independently, including some or other domains depending on the author [3]. So it should be reasonable to ask ourselves if using an adaptive behavior assessment we obtain the information that we are searching for ADL. Although a wide range of instruments to measure BADL performance in Spanish schoolchildren exists, we have found some issues that can influence the final occupational diagnosis: some of them are translated into Spanish, but no cultural adaptation information was found (e.g., Vineland Adaptive Behavior Scales II and PEDI-CAT); others measure daily skills, but are focused on early childhood (e.g., Portage Guide to Early Education, Merrill–Palmer-Revised Scales of Development, and Battelle Developmental Inventory), not covering the full school age range; and last, most of the tools come from an adaptive behavior point of view, not from an ADL-centered perspective (see Table 2).

To the best of our knowledge, a comprehensive tool to evaluate a full range of BADL is needed, especially to help OT practitioners in their clinical interventions. It is not our intention to develop a diagnostic measurement, but to describe as broadly as possible the performance of the pediatric population in BADL. We also aim to obtain screening information about the cognitive processes that influence BADL. The study aims to present a new tool to evaluate BADL performance in Spanish schoolchildren from 6 to 12 years of age showing its psychometric properties.

## 2. Materials and Methods

### 2.1. Participants

Inclusion criteria were being from 6 to 12 years old with typical development and providing a written informed consent form signed by legal guardians to the investigators. 375 schoolchildren of both genders participated in the study (177 boys, 47.2%, and 198 girls, 52.8%). Their age ranged from 6 to 12 years old (M = 8.5 years, SD = 2.1 for the total; M = 8.4 years, SD = 2 for boys; and M = 8.6 years, SD = 2.1 for girls). A probabilistic polietapic sample design was used, recruited through regular schools and sporting events in the community of Extremadura (Spain).

### 2.2. Instruments

The material used was the Activities of Daily Living Evaluation in Schoolchildren (ADL-E), a new tool created for Spanish schoolchildren, which covers a wide range of BADL. The instrument is made up of 4 different scales: 3 for measuring BADL (Eating, Personal Hygiene, and Getting Dressed) and 1 scale for cognitive aspects that influence BADL performance. The final version has 84 items + 6 qualitative items for girls (Table 3).

The Eating scale has 20 items. Aspects related to choosing, manipulating, chewing food, or drinks and keeping good manners during mealtime are explored. The Personal Hygiene scale is composed of 29 items. They help to obtain information about grooming, washing, brushing, showering, toileting, using supplies and cosmetics, and bladder and bowel control. Within this scale, 6 extra qualitative items about hairstyle and menstruation management for girls were included. The Getting Dressed scale has 19 items and explores aspects about choosing and adjusting clothes and accessories, and the dressing and undressing sequence, including shoes. The General Functioning scale has 18 items, which provide information about cognitive aspects related to EF and SR.

The ADL-E must be completed by conducting a family–therapist interview with caregivers, which must provide answers based on the behaviors they observe in their children. Each item must be answered by marking one of the four response options (Table 4). Therapists should obtain evidence/s that parents’ answers are as detailed as possible, so blank spaces for observations are provided. 

### 2.3. Procedure

**Figure 1:**
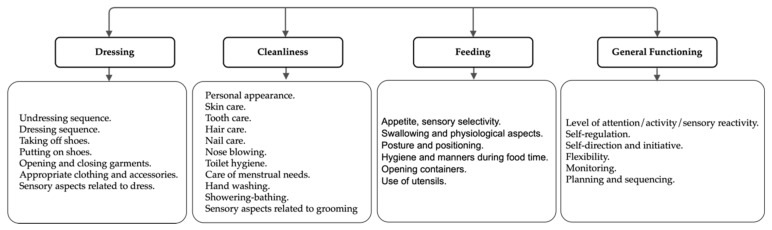
Initial categorization of the items.

After reviewing available instruments, it was decided to create a new one which meets several conditions: being culturally adapted, suitable for schoolchildren (6–12 years), and with an ADL-centered perspective. Cognitive items that influence occupational performance were added to help OT practitioners in their clinical intervention or recommending a thorough evaluation of dysfunction suspicion. A group of experts in the fields of psychology and OT created a preliminary version using the OT practice framework [1] and their clinical experience as the baseline (Figure 1). 

**Figure 2:**
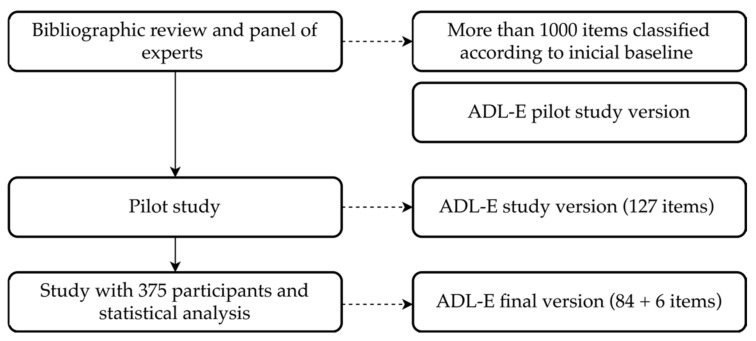
Activities of Daily Living Evaluation in Schoolchildren (ADL-E) creation procedure.

A pilot study was carried out with 15 families obtaining the version used for this study. Once administered to the sample, the items were analyzed by the group of experts, discarding those which did not fit on the theoretical model. This process resulted in the final version of the ADL-E as shown in Figure 2. This protocol adhered to the updates of the Declaration of Helsinki [47], and it was approved by the Committee on Biomedical Ethics of the University of Extremadura (198/2019).

### 2.4. Statistics

Microsoft Office^TM^ Excel v.16, FACTOR v.10.10.02, and IBM^TM^ SPSS v.25 were used for data analysis. An exploratory factor analysis (EFA) was carried out to find out the internal structure of every scale and to check the factorial weights of every item. Before the EFA, the two necessary conditions were verified: adjustment of the data to the normal curve and adequate sample adjustment indicators through the Kaiser–Meyer–Olkin (KMO) and Bartlett’s sphericity tests [48,49].

Due to the ordinal nature of the data, the EFA was used for the calculation of polychoric correlations using the robust unweighted least squares method (RULS) for the extraction of factors with oblique rotation because we started from the premise that factors were correlated. Items with factorial weights < .30 were maintained, and those with lower values were deleted [50]. This procedure, carried out with FACTOR, also allowed us to explore the goodness-of-fit data for each of the factor solutions [51,52]. This semiconfirmatory factor analysis of the items (SCFA) is suitable to prevent errors such as the ones of the “Little Jiffy” approach in psychometry [53,54,55].

To evaluate the goodness-of-fit of the model, the following were used: a) the chi-squared probability taking as appropriate non-significant values (*p* > .05); b) the comparative fit index (CFI) and the non-normed fit index (NNFI) considering > .90 as an indicator of good fit; c) the root mean square error of approximation (RMSEA), considering values < .06 acceptable; and e) the root mean square of residuals (RMSR) considering values < .05 acceptable [49,56]. Thus, the study of the EFA and the matrix of correlations between the factors together with the bibliographic review provided elements of judgment for the establishment of a theoretical model of relationships between the different scales and their factors.

To find out the internal consistency of the ADL-E, the ordinal alpha was used, which is an alternative to the Cronbach’s Alpha more accurate with Likert scale responses, including ordinal items. Preferred values are between < .80 and >.90, but .70 is considered acceptable [57,58].

## 3. Results

After performing the analyses, the items from the ADL-E were reduced from 124 (study version) to 84 + 6 qualitative items in the final version. The 4 scales are easily explained by a conceptual model with 12 factors. Each scale with its factors is explained below.

### 3.1. Eating Scale

The Eating scale aims to obtain information about choosing, manipulating, chewing food, or drinks, keeping good manners, and other aspects during mealtime. We found an interpretable solution with 4 factors (Table 5): Manual Dexterity (6 items), Proprioception (4 items), Oral Sensitivity (3 items), and Good Manners (7 items). The Manual Dexterity factor consists of the items related to the use of tools to open or manipulate food and drinks with manual or bimanual coordination requirements. The items in Proprioception are about the correct application of strength to manage food, drinks, or containers. In the Oral Sensitivity factor, the items are about sensory processing of food. The Good Manners during Mealtime factor groups items into several categories: cognitive, attentional, executive, sensory, motor, behavioral, and cultural ones. For example, a child who cannot stay seated during mealtime can have inhibition issues (executive), postural problems (sensory or motor), or he/she may not be interested in food (behavioral). Despite this, we named it Good Manners, because they all are considered correct behaviors in our society.

For the KMO test, a value of .71, and for the Bartlett’s test, *p* <.0.001 were found, both considered good to perform the EFA. Initially, this scale was formed by 28 items, but 8 items did not reach the weight of < .30, so they were not kept (Table 6). Thus, a total of 20 items form this scale in the final version of the instrument.

### 3.2. Personal Hygiene Scale

The Personal Hygiene scale aims to obtain information about taking care of oneself. We found an interpretable solution with 4 factors (Table 7): Hygiene and Grooming (18 items), Toileting Needs Communication (2 items), Bladder and Bowel Control (4 items), and Showering (5 items). In the Hygiene and Grooming factor, information about hair, skin, and nail care, use of cosmetics, nose-blowing, washing hands, brushing teeth, and toilet management, but also about keeping everything clean and caring about having a good appearance is grouped. Factors 2 and 3 are about Bladder and Bowel Control: the first one is about being able to communicate toilet needs, and the second one is about being aware of these needs. Factor 4 contains all the items related to showering/bathing.

For the KMO test, a value of .921, and for the Bartlett’s test, *p* <.0.001 were found, both considered good to perform the EFA. Initially, this scale was formed by 42 items, but 7 items did not reach the weight of < .30, so they were not kept (Table 8). The 6 qualitative items for girls were not included in the EFA. Thus, a total of 29 + 6 items forms this scale in the final version of the instrument.

### 3.3. Getting Dressed Scale

The Getting Dressed scale aims to obtain information about dressing tasks. We found an interpretable solution with 2 factors (Table 9): Independent Dressing Tasks (13 items) and Full Dressing (4 items). In the Independent Dressing Tasks factor, all the items are related to specific and individual tasks needed for dressing (accessories, zippers). These can be tasks with essential cognitive functions (choosing and taking care of clothes) or about manual dexterity and praxis (fastening and adjusting clothes and accessories). In the Full Dressing factor, the items are about the complete activity of getting dressed or undressed.

For the KMO test, a value of .92, and for the Bartlett’s test, *p* < .0.001 were found, both considered good to perform the EFA. Initially, this scale was formed by 30 items, but 13 items did not reach the weight of < .30, so they were not kept (Table 10). Thus, a total of 17 items form this scale in the final version of the instrument.

### 3.4. General Functioning Scale

The General Functioning scale aims to obtain information about the cognitive aspects that can influence BADL performance. We found an interpretable solution with 2 factors (Table 11): Executive Function (8 items) and Self-Regulation (10 items). The Executive Function factor contains several subprocesses related to planning, sequencing, keeping focused on the task, time control, and solving problems. In the Self-Regulation factor, all the items are about self-control and being able to manage thoughts, attention, feelings, and responses related to stimuli.

For the KMO test, a value of .65, and for the Bartlett’s test, *p* < .0.001 were found, both considered good to perform the EFA. Initially, this scale was formed by 19 items, but 1 item, "Asks for help when necessary in his/her daily life activities", did not reach < .30, so it was not kept.

### 3.5. Correlations Between Factors

Correlations between factors were also explored, as showed in Table 12 [59].

High correlations were found between Dre-F1/ Eat-F1, GF-F2/ Ea-F4, PH-F4/PH-F1, Dre-F1/ PH-F1, and GF-F2/ PH-F1. Moderate correlations were also found between Eat-F2/Eat-F1, Eat-F4/Eat-F1, PH-F1/Eat-F1, PH-F4/Eat-F1, GF2/ Eat-F1, Eat-F4/Eat-F2, PH-F1/ Eat-F2, Dre-F1/Eat-F2, PH-F1/Eat-F4, Dre-F1/Eat-F4, Dre-F1/ PH-F4, and GF-F2/Dre-F1.

### 3.6. Goodness-of-Fit Indices

As mentioned previously, the FACTOR software explores the goodness-of-fit data (see Table 13). All the indices are acceptable [49,56].

### 3.7. Reliability

To find out the internal consistency of the ADL-E, ordinal alpha was used (Table 14). As mentioned previously, the preferred values are < .80 and > .90, but < .70 is considered acceptable.

## 4. Discussion

Our main contribution is to present a new tool to assess BADL performance in Spanish schoolchildren. To our knowledge, no valid and reliable tool that covers the complete school age range and is BADL-centered to capture the occupational performance exists. Finally, 4 scales form the ADL-E: Eating, Personal Hygiene, Getting Dressed + General Functioning. A total of 12 factors offer a conceptual model we think is relevant: the Eating scale is formed by 4 factors: Manual Dexterity while Eating (6 items), Proprioception (4 items), Oral Sensitivity (3 items), and Good Manners (7 items); the Personal Hygiene scale is formed by 4 factors: Hygiene and Grooming (18 items), Toileting Needs Communication (2 items), Bladder and Bowel Control (4 items), and Showering (5 items); the Getting Dressed scale is formed by 2 factors: Independent Dressing Tasks (13 items) and Full Dressing (4 items); and the General Functioning scale is formed by 2 factors: Executive Function (8 items) and Self-Regulation (10 items). This structure is different and more accurate than the one presented by other instruments: for example, Adaptive Behavior Assessment System II (ABAS II) groups all the BADL items in a single section called Self-Care; the Inventory for Client and Agency Planning (ICAP) joins all the BADL items in the Personal Life Skills section; Vineland Adaptive Behavior Scales II (VABS II)—in the Daily Living Skills Domain—Personal; the Merrill–Palmer-Revised Scales of Development (MP-R)—in Adaptive Behavior and Self-Care, and the Portage Guide to Early Education—in the Self-Care section. More specific, but not BADL-centered, are the Battelle Developmental Inventory (BDI) with the following sections in the Adaptive Scale: Attention, Mealtime, Getting Dressed, Personal Responsibility, and Hygiene; and the CALS, with Socialization, Mealtime, Hygiene and Grooming, Toileting, Getting Dressed, Health Care (this is an IADL), and Sexuality. The Computer Adaptive Test (PEDI-CAT), which is the closest to the ADL-E, is formed by 4 domains: Daily Activities (including BADL and IADL), Mobility, Social/Cognitive, and Responsibility, but it is not culturally validated in Spain.

Related to correlations between factors, several facts need to be considered. Manual Dexterity in the Eating factor has a strong correlation with the Independent Dressing Tasks factor, which can be explained by the critical motor requirements of that kind of tasks/activities. Good Manners during Mealtime and Self-Regulation also have a strong correlation, which also can be explained by the need to inhibit and manage behaviors to keep seated following cultural customs. The Hygiene and Grooming factor also has a high correlation with Showering (some of the steps in hygiene are common in showering), Independent Dressing Tasks (also requires developed motor skills) and the Self-Regulation factor (high demand of self-control while performing these tasks). The ADL-E shows good psychometric properties, both in validity and reliability (internal consistency).

Continuing with the ADL-E structure and exploring the number of items, the final number is 84 + 6 qualitative items for girls, so the time to complete the interview should be 45–90 minutes, which can be reasonable and accessible for OT practitioners and other professionals. Some of the reviewed tools have a much lower number of items, so perhaps they do not provide enough information (e.g., the ICAP with 21 items, ABAS II with 24, or the Vineland with 41), and others had a much higher number, so maybe the professionals have not got the time to properly administer them (e.g., the CALS with 814 items). The ADL-E can be a useful tool to help therapists to make clinical decisions. As mentioned previously, OT practitioners need assessments to help them to characterize the BADL performance, because it is one of the most demanded interventions within their scope. The ADL-E can also be useful for educational professionals and families to have a reference about the right acquisition of BADL abilities by their students or children.

This research had some limitations. The sample was recruited in the community of Extremadura. We tried to establish a development trajectory for the BADL considered universal, but maybe social and cultural differences in this kind of activities should be more deeply checked. Another important aspect is that the information is completed through caregivers. Although instruments completed by families are considered to be valid tools [60], some authors warn us to be careful, because parents could overestimate or underestimate development of their children [61,62]. We also need to improve concurrent validity using well-established tools.

Concerning future lines of research, we have several appreciations to do. In the occupational therapy, tools to measure BADL are necessary. For physically disabled children, we have specific tools to assess the issues they usually have with their ADL: for example, the Pediatric Evaluation of Disability Inventory (PEDI) [63], or the Functional Independence Measure for Children (WeeFIM) [64], which are very focused on physical problems with mobility BADL. So, an important future line should be using the ADL-E with specific populations with cognitive disorders or impairments, because to the best of our knowledge, there is blank space in this area. For example, children diagnosed with neurodevelopmental disorders suffering from alterations or delays in the development of functions related to the maturation of the central nervous system that causes difficulties to adapt to the environment [65] should be an interesting group to explore. Within this group, autism spectrum disorders (ASD), intellectual disability (ID), attention deficit and hyperactivity disorders (ADHD), motor disorders, specific learning disorder, communication disorders, etc. are included. Some authors have found differences in ADL profiles between these populations: worse performance in ASD children in hygiene or dressing than in the ID population [66] or worse performance in dressing, personal hygiene, and eating skills, including postural control and fine motor skills, in children with developmental coordination disorder compared with normally developed children [11], so it should be interesting to try to find specific BADL profiles.

## 5. Conclusions

The ADL-E is a practical and easy-to-apply tool which assesses BADL (Eating, Personal Hygiene, and Getting Dressed) in Spanish schoolchildren aged 6–12. The ADL-E also offers monitoring of the influence of EF and SR during these activities, showing good psychometric properties in both validity and reliability.

## Figures and Tables

**Table 1:** Aspects of the domain of occupational therapy [1].

Occupations	Client Factors	Performance Skills	Performance Patterns	Context and Environment
Basic ADL Instrumental ADL Rest and sleep Education Work Play Leisure Social participation	Values, beliefs, and spirituality Body functions Body structures	Motor Process Social interaction	Habits Routines Rituals Roles	Cultural Personal Physical Social Temporal Virtual

ADL: activities of daily living.

**Table 2:** Summary of BADL and adaptative behavior measurements for Spanish children.

Tool	Spanish Translated/ Validated	Full 6–12 Years Age Range	BADL-Centered Assessment
Adaptive Behavior Assessment System II – ABAS II [31,32]	yes/yes	yes	no
Checklist of Adaptive Living Skills – CALS [33,34]	yes/yes	yes	no
Inventory for Client and Agency Planning – ICAP [35,36]	yes/yes	yes	no
Vineland Adaptive Behavior Scales II – VABS II [37]	yes/no	yes	no
Pediatric Evaluation of Disability Inventory—Computer Adaptive Test – PEDI-CAT [38]	yes/no	yes	yes
Supports Intensity Scale – SIS [39,40]	yes/yes	yes	no
Battelle Developmental Inventory – BDI [41,42]	yes/yes	no	no
Merrill–Palmer-Revised Scales of Development – MP-R [43,44]	yes/yes	no	no
Portage Guide to Early Education [45,46]	yes/yes	no	no

BADLs: basic activities of daily living.

**Table 3:** Qualitative ADL-E items for girls on the Personal Hygiene scale.

1. She can do hairstyles with bobby pins, make pigtails, etc.
2. She understands what menstruation is.
3. She knows how to apply a pad.
4. The pad is changed at an appropriate frequency, without too many accidents.
5. She leaves used pads inside containers.
6. She washes her hands after changing the pad.
ADL-E: Activities of Daily Living Evaluation in Schoolchildren.

**Table 4:** Activities of Daily Living Evaluation in Schoolchildren (ADL-E) response options.

Response Options	Explanation
Always	The child can perform the task/activity independently. It always happens.
Sometimes	The child is learning to do the task/activity. The child needs help (visual, verbal, or physical) from an adult. It happens sometimes.
Never	The child cannot perform the task/activity. An adult does the essential parts of the activity’s tasks. It never happens.
Not known; no opportunity	The informant cannot answer. The child has never faced that situation.

**Table 5:** Eating scale factorial solution.

Item Number	Item	Factorial Weight
**Factor 1: Manual Dexterity while Eating.**		
19	He/she uses tools to open containers (e.g., a can opener).	0.393
24	He/she uses a knife to spread.	0.780
25	He/she uses a knife to cut food.	0.927
26	He/she uses several cutleries at the same time in a coordinated way (e.g., a knife and a fork).	0.860
27	He/she can serve food from a bowl or tray.	0.770
28	He/she chooses the right cutlery.	0.447
**Factor 2: Proprioception.**		
11	He/she licks foods like ice cream or candy.	0.383
16	He/she opens containers with pressure plugs.	0.545
17	He/she unscrews screw caps.	0.800
21	He/she sips on a straw.	0.302
**Factor 3: Oral Sensitivity.**		
3	He/she is reluctant to try new foods.	0.825
4	He/she is unwilling to eat food with certain textures (purees, crunchy...).	0.875
5	He/she shows disgust when certain foods are within his/her mouth.	0.744
**Factor 4: Good Manners during Mealtime.**		
2	He/she only puts edible substances into his/her mouth.	0.331
7	He/she tests the food carefully to check its temperature.	0.470
9	He/she chews with his/her mouth closed.	0.543
12	He/she maintains a proper posture during mealtime.	0.795
13	He/she keeps seated at the table during mealtime.	0.727
14	He/she uses napkins properly.	0.628
15	He/she tries to maintain good manners during mealtime.	0.848

**Table 6:** Deleted items from the Eating scale.

Item Number	Item
1	He/she asks for food or water when hungry or thirsty.
6	If necessary, he/she collaborates, taking his/her medication.
8	He/she drinks liquids without spilling from the glass or the bottle.
10	He/she chews food until crushed before swallowing.
18	He/she can open wrappers.
20	He/she can eat pieces of food with his/her hands.
22	He/she can use a spoon without spilling food.
23	He/she can prick food with a fork.

**Table 7:** Personal Hygiene scale factorial solution.

Item Number	Item	Factorial Weight
**Factor 1: Hygiene and Grooming.**		
7	In the bathroom, he/she acceptably get clean with toilet paper.	0.381
9	When he/she is done using the toilet, he/she lowers the lid and pulls the chain.	0.469
10	He/she washes his/her hands after using the toilet.	0.541
11	In the bathroom, he/she cares about his/her privacy.	0.493
12	He/she collaborates using cologne or moisturizer.	0.448
13	He/she keeps his/her nails clean.	0.662
14	He/she brushes his/her hair.	0.460
16	He/she checks his/her appearance before leaving home.	0.418
17	He/she brushes his teeth after eating without being told by an adult.	0.638
19	He/she brushes most or all areas of his/her mouth.	0.636
20	He/she spits into the wash when brushing his/her teeth.	0.835
21	After brushing his/her teeth, he/she checks there are no traces of paste left in his/her mouth or face.	0.591
22	He/she leaves the sink clean and picks up everything after brushing.	0.506
24	He/she is aware when he/she needs to wipe his/her nose.	0.590
25	He/she can blow his nose.	0.494
26	He/she can adjust the water pressure.	0.492
28	When washing his/her hands, soap and water are given to full hands.	0.602
29	When washing his/her hands, he/she uses an adequate amount of soap.	0.523
**Factor 2: Toileting Needs Communication.**		
5	He/she communicates his/her need to go to the bathroom.	0.508
6	He/she warns an adult when he/she has had an accident (peed or pooped).	0.777
**Factor 3: Bladder and Bowel Control.**		
1	Usually, he/she stays poopless at night.	0.673
2	Usually, he/she stays dry at night, without peeing.	0.600
3	He/she keeps clean during the day, without pooping him/herself.	0.723
4	He/she keeps dry during the day, without peeing him/herself.	0.927
**Factor 4: Showering.**		
32	In the shower, he/she soaps up all over the body.	0.920
33	In the shower, he/she rinses until all foam is removed.	0.850
34	In the shower, he/she lathers and rinses his/her intimate parts carefully.	0.846
35	In the shower, he/she uses the towel until he/she is relatively dry.	0.564
36	In the shower, he/she lathers his/her hair in an acceptable way.	0.780

**Table 8:** Deleted items from the Personal Hygiene scale.

Item Number	Item
8	He/she can lower or raise his/her clothes to use the toilet.
18	He/she brushes for at least one minute.
23	He/she is stressed while brushing teeth.
27	He/she can check and adjust the water temperature.
30	When he/she washes his/her hands, he/she wipes himself/herself completely dry.
31	He/she can wash his/her face.
37	He/she is stressed when nails or hair are cut.

**Table 9:** Getting Dressed scale factorial solution.

Item Number	Item	Factorial Weight
**Factor 1: Independent Dressing Tasks.**		
4	He/she can choose his/her clothing and accessories depending on the weather conditions.	0.580
5	He/she makes sure that the label of the clothes is in the right place.	0.601
6	He/she distinguishes when his/her clothes are clean or dirty.	0.491
8	He/she can put his/her socks properly.	0.739
14	He/she undresses completely, including using zippers on garments.	0.447
15	He/she takes off his/her clothes, leaving them on the right side (label inside).	0.468
16	He/she can put on a coat or an open garment.	0.360
20	He/she puts on accessories (e.g., gloves, scarf, hat, etc.).	0.518
22	He/she clasps snap buttons (click type).	0.718
24	He/she can zip clothes up.	0.601
26	He/she opens buttons.	0.775
27	He/she can undo his/her shoes’ lacing.	0.807
28	He/she can tie a knot in his/her shoes.	0.715
**Factor 2: Full Dressing.**		
17	He/she can put on stretching pants.	0.856
18	He/she can put on a T-shirt or an upper garment.	0.840
19	He/she can get dressed without help (not including closures).	0.738
30	He/she can get dressed without help (including closures and accessories).	0.520

**Table 10:** Deleted items from the Getting Dressed scale.

Item Number	Item
1	Labels or certain fabrics bother him/her.
2	He/she does not seem to notice that garments are misplaced.
3	He/she always wants to wear the same clothing.
7	He/she can remove socks.
9	He/she can put footwear on his/her feet.
10	He/she places a shoe on the right foot.
11	He/she can remove shoes without fasteners.
12	He/she can remove shoes with fasteners.
13	He/she can remove simple garments without closures (pants, underwear).
21	He/she opens and closes Velcro fasteners.
23	He/she can zip up and down.
25	He/she can unbutton.
29	He/she can tie his/her shoes.

**Table 11:** General Functioning scale factorial solution.

Item Number	Item	Factorial Weight
**Factor 1: Executive Function.**		
2	He/she begins his/her activities of daily life in a reasonable time from the adult’s direction.	0.540
3	In general, he/she can perform his/her activities of daily living without the help of an adult.	0.556
6	He/she persists in their activities of daily life although he/she finds difficulties (e.g., while he is cutting a steak).	0.519
10	He/she finishes his/her activities of daily living at an appropriate time, not too early, not too late (e.g., washing hands).	0.599
11	He/she becomes aware of the mistakes he/she makes in his/her daily life activities (e.g., if the lacing of his/her shoe gets loose).	0.523
12	He/she tries to solve problems while performing an activity (e.g., he/she knows what to do if the toothpaste is over).	0.649
13	He/she can perform his/her daily activities without unnecessary stops.	0.662
15	He/she performs his/her daily activities in a logical order (e.g., putting on the underwear before a garment).	0.688
**Factor 2: Self-Regulation.**		
4	He/she gets frustrated quickly when he/she cannot perform some of his/her daily activities.	0.363
5	He/she has more tantrums than expected for a child of his/her age.	0.502
7	He/she finds it difficult to get adapted to changes in the environment.	0.769
8	It is difficult for him/her to assimilate changes in his/her routine.	0.801
9	It is difficult for him/her to stop performing one activity to move on to another, especially if he/she enjoys the activity he/she is doing.	0.526
14	He/she has difficulties performing activities of daily living with two or more steps (e.g., brushing teeth or getting fully dressed).	0.581
16	He/she often leaves his/her activities of daily living unfinished (e.g., when he/she dries his/her hands, they remain wet).	0.591
17	He/she loses his/her attention performing his/her daily activities if there is any external noise.	0.598
18	Sometimes he/she spins or rocks excessively, which makes it difficult to perform his/her daily activities.	0.595
19	He/she does not perform his/her activities of daily life properly due to excessive movement.	0.545

**Table 12:** Correlations between factors.

	Eat-F1	Eat-F2	Eat-F3	Ea-F4	PH-F1	PH-F2	PH-F3	PH-F4	Dre-F1	Dre-F2	GF-F1	GF-F2
**Eat-F1**	1											
**Eat-F2**	−0.43 **	1										
**Eat-F3**	0.05	−0.17 **	1									
**Eat-F4**	−0.42 **	0.40 **	−0.08	1								
**PH-F1**	−0.55 **	0.40 **	−0.07	0.58 **	1							
**PH-F2**	0.03	0.06	0.09	−0.01	−0.02	1						
**PH-F3**	−0.07	0.06	−0.10 *	0.02	0.04	0.02	1					
**PH-F4**	0.46 **	−0.35 **	0.01	−0.37 **	−0.61 **	−0.06	−0.03	1				
**Dre-F1**	−0.61 **	0.44 **	−0.11 *	0.44 **	0.60 **	0.03	0.09	−0.48 **	1			
**Dre-F2**	−0.15 **	0.10 *	−0.14 **	0.07	0.06	−0.08	0.27 **	−0.15 **	0.25 **	1		
**GF-F1**	0.18 **	−0.17 **	0.24 **	−0.33 **	−0.32 **	0.08	−0.06	0.19 *	−0.23 **	0.08	1	
**GF-F2**	−0.45 **	0.36 **	−0.17 **	0.61 **	0.59 **	0.03	0.02	−0.38 **	0.55 **	0.11 *	−0.36 **	1

* Correlation is significant at the 0.05 level (2-tailed). ** Correlation is significant at the 0.01 level (2-tailed). Eat-F1: Eating scale—factor 1; PH-F1: Personal Hygiene scale—factor 1; Dre-F1: Getting Dressed scale—factor 1; GF-F1: General Functioning scale—factor 1. The same is applied to factors 2, 3, and 4.

**Table 13:** ADL-E goodness-of-fit indices.

ADL-E scales	Results	Cutoff
**Eating scale**		
*p* (χ2)	*p* = 0.18	> 0.05
CFI	0.99	> 0.90
NNFI	0.99	> 0.90
RMSEA	0.03	< 0.06
RMSR	0.05	< 0.08
**Personal Hygiene scale**		
*p* (χ2)	*p* = 0.01	> 0.05
CFI	0.99	> 0.90
NNFI	0.99	> 0.90
RMSEA	0.02	< 0.06
RMSR	0.06	< 0.08
**Getting Dressed scale**		
*p* (χ2)	*p* = 0.24	> 0.05
CFI	0.98	> 0.90
NNFI	0.99	> 0.90
RMSEA	0.05	< 0.06
RMSR	0.08	< 0.08
**General Functioning scale**		
*p* (χ2)	*p* = 0.00	> 0.05
CFI	0.98	> 0.90
NNFI	0.99	> 0.90
RMSEA	0.04	< 0.06
RMSR	0.07	< 0.08

*p* (χ2): chi-squared probability; CFI: comparative fit index; NNFI: non-normed fit index, RMSEA: root mean square error of approximation; RMSR: root mean square of residuals.

**Table 14:** Internal consistency of the ADL-E.

ADL-E Factors	α
**Eating scale**	
Manual Dexterity while Eating.	0.85
Proprioception.	0.70
Oral Sensitivity.	0.85
Good Manners during Mealtime.	0.81
**Personal Hygiene scale**	
Hygiene and Grooming.	0.85
Toileting Needs Communication.	0.70
Bladder and Bowel Control.	0.85
Showering.	0.81
**Getting Dressed scale**	
Independent Dressing Tasks.	0.88
Full Dressing.	0.82
**General Functioning scale**	
Executive Function	0.81
Self-Regulation	0.84

## References

[1] American Occupational Therapy Association (2014). Occupational therapy practice framework: Domain and process 3a ed. Am. J. Occup. Ther..

[2] Moruno Miralles P., Romero Ayuso D.M. (2006). Actividades de la Vida Diaria.

[3] Romero D.M. (2007). Actividades de la vida diaria. An. Psicol..

[4] American Occupational Therapy Association (2002). Occupational Therapy Practice Framework: Domain and Process. Am. J. Occup. Ther..

[5] American Occupational Therapy Association (2008). Occupational Therapy Practice Framework: Domain and process 2nd Edition. Am. J. Occup. Ther..

[6] Jefatura del Estado (2006). Ley 39/2006, de 14 de diciembre, de Promoción de la Autonomía Personal y Atención a las Personas en Situación de Dependencia.

[7] Schalock R.L., Verdugo M.A. (2003). Quality of Life for Human Service Practitioners.

[8] Organización Mundial de la Salud (2001). Clasificación Internacional del Funcionamiento de la Discapacidad y de la Salud.

[9] Gantschnig B.E., Fisher A.G., Page J., Meichtry A., Nilsson I. (2015). Differences in activities of daily living (ADL) abilities of children across world regions: A validity study of the assessment of motor and process skills: ADL differences in children across world regions. Child Care Health Dev..

[10] Günal A., Bumin G., Huri M. (2019). The Effects of Motor and Cognitive Impairments on Daily Living Activities and Quality of Life in Children with Autism. J. Occup. Ther. Sch. Early Interv..

[11] Summers J., Larkin D., Dewey D. (2008). Activities of daily living in children with developmental coordination disorder: Dressing, personal hygiene, and eating skills. Hum. Mov. Sci..

[12] Beaussart M.-L., Barbarot S., Mauger C., Roy A. (2018). Systematic Review and Meta-analysis of Executive Functions in Preschool and School-Age Children with Neurofibromatosis Type 1. J. Int. Neuropsychol. Soc..

[13] Gray-Burrows K., Taylor N., O’Connor D., Sutherland E., Stoet G., Conner M. (2019). A systematic review and meta-analysis of the executive function-health behaviour relationship. Health Psychol. Behav. Med..

[14] Papazian O., Alfonso I., Luzondo R.J. (2006). Trastornos de las funciones ejecutivas. Rev. Neurol..

[15] Martos Pérez J., Paula Pérez I. (2011). Una aproximación a las funciones ejecutivas en el trastorno del espectro autista. Rev. Neurol..

[16] Collette F., Hogge M., Salmon E., Van der Linden M. (2006). Exploration of the neural substrates of executive functioning by functional neuroimaging. Neuroscience.

[17] Miyake A., Friedman N.P., Emerson M.J., Witzki A.H., Howerter A., Wager T.D. (2000). The Unity and Diversity of Executive Functions and Their Contributions to Complex “Frontal Lobe” Tasks: A Latent Variable Analysis. Cogn. Psychol..

[18] Tirapu Ustárroz J., Bausela Herreras E., Cordero Andrés P. (2018). Modelo de funciones ejecutivas basado en análisis factoriales en población infantil y escolar: Metaanálisis. Rev. Neurol..

[19] Estévez González A., García Sánchez C., Barraquer i Bordas L. (2000). Los lóbulos frontales: El cerebro ejecutivo. Rev. Neurol..

[20] Ismael N., Lawson L.M., Hartwell J. (2018). Relationship Between Sensory Processing and Participation in Daily Occupations for Children with Autism Spectrum Disorder: A Systematic Review of Studies That Used Dunn’s Sensory Processing Framework. Am. J. Occup. Ther..

[21] Elbasan B., Kayıhan H., Duzgun I. (2012). Sensory integration and activities of daily living in children with developmental coordination disorder. Ital. J. Pediatr..

[22] Hofmann W., Schmeichel B., Baddeley A. (2012). Executive functions and self-regulation. Trends Cogn. Sci..

[23] McMahon K., Anand D., Morris-Jones M., Rosenthal M.Z. (2019). A Path from Childhood Sensory Processing Disorder to Anxiety Disorders: The Mediating Role of Emotion Dysregulation and Adult Sensory Processing Disorder Symptoms. Front Integr. Neurosci..

[24] Martini R., Cramm H., Egan M., Sikora L. (2016). Scoping Review of Self-Regulation: What Are Occupational Therapists Talking About?. Am. J. Occup. Ther..

[25] Murray D., Rosanbalm K., Hamoudi A. (2015). Self-Regulation and Toxic Stress: Foundations for Understanding Self-Regulation from an Applied Developmental Perspective.

[26] DeGangi G.A. (2017). Pediatric Disorders of Regulation in Affect and Behavior: A Therapist’s Guide to Assessment and Treatment.

[27] Howard S.J., Vasseleu E. (2020). Self-Regulation and Executive Function Longitudinally Predict Advanced Learning in Preschool. Front. Psychol..

[28] Howard S.J., Vasseleu E., Batterham M., Neilsen-Hewett C. (2020). Everyday Practices and Activities to Improve Pre-school Self-Regulation: Cluster RCT Evaluation of the PRSIST Program. Front. Psychol..

[29] Payne S. (2016). Standardised Tests: An Appropriate Way to Measure the Outcome of Paediatric Occupational Therapy. Br. J. Occup. Ther..

[30] Piernik-Yoder B., Beck A. (2012). The Use of Standardized Assessments in Occupational Therapy in the United States. Occup. Ther. Health Care.

[31] Harrison P.L., Oakland T. (2003). ABAS II.

[32] Montero D., Fernández-Pinto I. (2013). ABAS® II: Sistema Para la Evaluación de la Conducta Adaptativa: Manual.

[33] Bruininks R.L. (1991). Adaptive Living Skills Curriculum: Manual.

[34] Morreau L.E., Bruininks R.H., Montero Centeno D., Universidad de Deusto, Instituto de Ciencias de la Educación (2006). Inventario de Destrezas Adaptativas (CALS): Manual.

[35] Bruininks R.K., Hill B.K., Weatherman R.F., Woodcock R.W. (1986). Inventary for Client and Agency Planning.

[36] Montero D. (1999). Evaluación de la conducta adaptativa en personas con discapacidades.

[37] Sparrow S.S., Cichetti D.V., Balla D.A. (2005). Vineland Adaptive Behavior Scales.

[38] Haley S., Coster W., Ludlow L., Haltiwanger J., Andrellos P. (1992). Pediatric Evaluation of Disability Inventory (PEDI). Development, Standardization and Manual Administration.

[39] Thompson J.R., Verdugo M.A. (2007). Escala de Intensidad de Apoyos - SIS: Manual.

[40] Thompson J.R., Bryant B.R., Campbell E.M., Craig E.M., Hughes C.M., Rotholz D.A. (2004). Supports Intensity Scale User’s Manual.

[41] Cruz M.V.d.l., González Criado M., Newborg J. (1996). Battelle, Inventario de Desarrollo: Manual de Aplicación.

[42] Newborg J., Stock J.R., Wnek L., Guidubaldi J., Svinicki J. (1984). Battelle Developmental Inventory: Examiner’s Manual.

[43] Roid G.H., Sampers J. (2004). Merrill-Palmer-Revised Scales of Development.

[44] Sánchez F., Santamaría P., Fernández-Pinto I., Arribas D. (2011). Escalas de Desarrollo Merrill-Palmer Revisadas.

[45] Bluma S., Sherer M., Frohman A., Hilliard J. (1978). Guía Portage de Educación Preescolar.

[46] Shearer M.S., Shearer D.E. (1972). The Portage Project: A model for early childhood education, Exceptional Children. Except Child.

[47] World Medical Association Declaración de Helsinki de la AMM—Principios éticos para las investigaciones médicas en seres humanos. https://www.wma.net/es/policies-post/declaracion-de-helsinki-de-la-amm-principios-eticos-para-las-investigaciones-medicas-en-seres-humanos/.

[48] Jöreskog K.G., Sörbom D. (1996). LISREL 8: User’s Reference Guide.

[49] Frías-Navarro M.D.F., Pascual-Soler M.P. (2012). Prácticas del análisis factorial exploratorio (AFE) en la investigación sobre conducta del consumidor y marketing. Suma Psicol..

[50] Beavers A.S., Lounsbury J.W., Richards J.K., Huck S.W., Skolits G.J., Esquivel S.L. (2013). Practical considerations for using exploratory factor analysis in educational research. PARE.

[51] Ferrando P.J., Lorenzo-Seva U. (2014). El análisis factorial exploratorio de los ítems: Algunas consideraciones adicionales. An. Psicol..

[52] Lorenzo-Seva U.F.P.J. (2013). FACTOR 9.2: A Comprehensive Program for Fitting Exploratory and Semiconfirmatory Factor Analysis and IRT Models. Appl. Psychol. Meas..

[53] Ferrando P.J., Lorenzo-Seva U. (2017). Program FACTOR at 10: Origins, development and future directions. Psicothema.

[54] Lloret S., Ferreres A., Hernández A., Tomás I. (2017). The exploratory factor analysis of items: Guided analysis based on empirical data and software. An. Psicol..

[55] Watkins M.W. (2018). Exploratory Factor Analysis: A Guide to Best Practice. J. Black Psychol..

[56] Ferrando P.J., Anguiano-Carrasco C. (2010). El análisis factorial como técnica de investigación en psicología. Pap. del Psicól..

[57] Gadermann A., Guhn M., Zumbo B.D., Michalos A.C. (2014). Ordinal Alpha. Encyclopedia of Quality of Life and Well-Being Research.

[58] Dominguez-Lara S. (2018). Fiabilidad y alfa ordinal. Actas Urol. Esp..

[59] Mukaka M.M. (2012). Statistics Corner: A guide to appropriate use of Correlation coefficient in medical research. Malawi. Med. J..

[60] Ministerio de Sanidad, Servicios Sociales e Igualdad (2014). Encuesta Nacional de Salud: España 2011/12. Salud Mental y Calidad de Vida en la Población Infantil.

[61] Van Gameren-Oosterom H.B.M., van Dommelen P., Schönbeck Y., Oudesluys-Murphy A.M., van Wouwe J.P., Buitendijk S.E. (2012). Prevalence of overweight in Dutch children with Down syndrome. Pediatrics.

[62] Pardo-Guijarro M.J., Martínez-Andrés M., Notario-Pacheco B., Solera-Martínez M., Sánchez-López M., Martínez-Vizcaíno V. (2015). Self-reports versus parental perceptions of health-related quality of life among deaf children and adolescents. J. Deaf. Stud. Deaf. Educ..

[63] Haley S.M., Coster W.J., Dumas H.M., Fragala-Pinkham M.A., Moed R. (2012). PEDI-CAT: Development, Standardization and Administration Manual.

[64] (1993). Guide for the Functional Independence Measure for Children (WeeFIM) of the Uniform Data System for Medical Rehabilitation, Version 4.0.

[65] American Psychiatric Association (2013). Diagnostic and Statistical Manual of Mental Disorders.

[66] Kilincaslan A., Kocas S., Bozkurt S., Kaya I., Derin S., Aydin R. (2019). Daily living skills in children with autism spectrum disorder and intellectual disability: A comparative study from Turkey. Res. Dev. Disabil..

